# Methyl 5-phenyl-1,2,3,4,4a,5,5a,13c-octahydro-6*H*-benzo[*f*]chromeno[3,4-*b*]indolizine-5a-carboxyl­ate

**DOI:** 10.1107/S1600536809026774

**Published:** 2009-07-18

**Authors:** E. Theboral Sugi Kamala, S. Nirmala, L. Sudha, S. Kathiravan, R. Raghunathan

**Affiliations:** a Department of Physics, Easwari Engineering College, Ramapuram, Chennai 600 089, India; b Department of Physics, SRM University, Ramapuram Campus, Chennai 600 089, India; c Department of Organic Chemistry, University of Madras, Guindy Campus, Chennai 600025, India

## Abstract

In the title compound, C_27_H_27_NO_3_, the pyrrolidine ring exhibits a twist conformation and the piperidine ring exhibits a chair conformation. The pyrrolidine ring makes dihedral angles of 54.47 (5), 51.50 (5) and 73.37 (6)° with the napthalene ring system and the tetra­hydro­pyran and phenyl rings, respectively. The structure is stabilized by intra­molecular C—H⋯O and C—H⋯N inter­actions.

## Related literature

For general background to the applications and biological activity of indolizine derivatives, see: Gubin *et al.* (1992 ▶); Gupta *et al.* (2003 ▶); Poty *et al.* (1994 ▶); Hema *et al.* (2003 ▶); Malonne *et al.* (1998 ▶); Medda *et al.* (2003 ▶). For puckering parameters, see: Cremer and Pople (1975 ▶). For asymmetry parameters, see: Nardelli (1983 ▶).
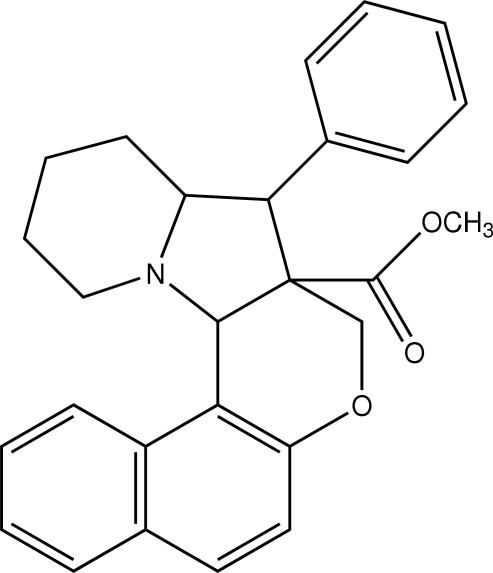


## Experimental

### 

#### Crystal data

C_27_H_27_NO_3_
*M* *_r_* = 413.50
Triclinic, 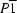

*a* = 9.4201 (3) Å
*b* = 10.6752 (3) Å
*c* = 11.0761 (3) Å
α = 78.262 (2)°
β = 77.911 (2)°
γ = 87.346 (2)°
*V* = 1066.34 (5) Å^3^
*Z* = 2
Mo *K*α radiation
μ = 0.08 mm^−1^
*T* = 293 K
0.30 × 0.20 × 0.15 mm

#### Data collection

Bruker Kappa APEXII diffractometer
Absorption correction: multi-scan (*SADABS*; Sheldrick, 1996 ▶) *T*_min_ = 0.975, *T*_max_ = 0.988
22685 measured reflections
4641 independent reflections
3461 reflections with *I* > 2σ(*I*)
*R*_int_ = 0.026

#### Refinement

*R*[*F* ^2^ > 2σ(*F* ^2^)] = 0.044
*wR*(*F* ^2^) = 0.122
*S* = 1.00
4641 reflections
281 parameters
H-atom parameters constrained
Δρ_max_ = 0.34 e Å^−3^
Δρ_min_ = −0.23 e Å^−3^

### 

Data collection: *APEX2* (Bruker, 2004 ▶); cell refinement: *APEX2* and *SAINT* (Bruker, 2004 ▶); data reduction: *APEX2* and *SAINT*; program(s) used to solve structure: *SHELXS97* (Sheldrick, 2008 ▶); program(s) used to refine structure: *SHELXL97* (Sheldrick, 2008 ▶); molecular graphics: *ORTEP-3* (Farrugia, 1997 ▶); software used to prepare material for publication: *PLATON* (Spek, 2009 ▶).

## Supplementary Material

Crystal structure: contains datablocks I, global. DOI: 10.1107/S1600536809026774/bt2988sup1.cif

Structure factors: contains datablocks I. DOI: 10.1107/S1600536809026774/bt2988Isup2.hkl

Additional supplementary materials:  crystallographic information; 3D view; checkCIF report

## Figures and Tables

**Table 1:** Hydrogen-bond geometry (Å, °)

*D*—H⋯*A*	*D*—H	H⋯*A*	*D*⋯*A*	*D*—H⋯*A*
C8—H8⋯O3	0.98	2.47	2.8240 (19)	101
C19—H19*B*⋯N1	0.97	2.55	2.885 (2)	100

**Fig. 1.**
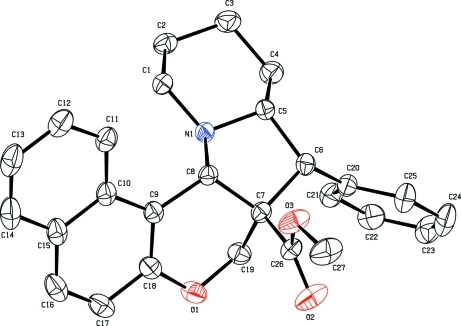
The molecular structure of (I) with 30% probability displacement ellipsoids.

**Fig. 2.**
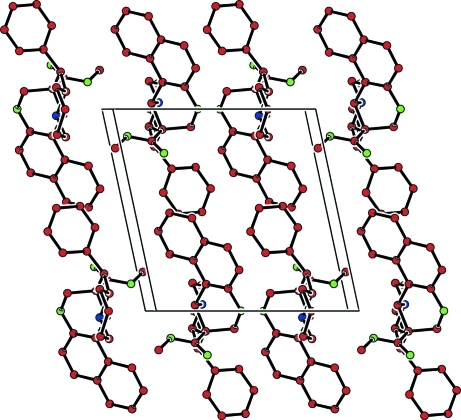
The packing of the molecules viewed along b axis.

## supplementary crystallographic information

### Comment

Indolizines, the nitrogen containing heterocyclic systems, are widely distributed in nature; in particular, indolizine derivatives are an important class of heterocyclic bioactive compounds with a wide range of applications, such as pharmaceutical drugs, potential central nervous system depressants, calcium entry blockers, cardiovascular agents, spectral sensitizers and novel dyes(Gubin *et al.*, 1992; Gupta *et al.*, 2003; Poty *et al.*, 1994; Hema *et al.*, 2003).Moreover indolizine derivatives have been found to possess a variety of biological activities such as antiinflammatory (Malonne *et al.*, 1998), antiviral (Medda *et al.*, 2003).

Fig 1 shows the *ORTEP*   plot of compound (I). Bond lengths and angles are comparable with other reported values.

In the molecule the pyrrolidine ring N1/C5/C6/C7/C8 exhibits *twist* conformation with assymetry parameters (Nardelli, 1983) ΔC_s_(N1) =23.66 (1)/ (C8) = 14.95 (1) and with the puckering parameters (Cremer and Pople, 1975) q2 = 0.4749 (1)Å and φ2 = 155.74 (2)°. The six membered ring N1/C1—C5 exhibits *chair* conformation with assymetry parameters ΔC_s_(N1) = 2.78 (1)/(C3) = 2.78 (1) and with the puckering parameters Q = 0.5788 (2) Å, Θ = 175.62 (2)° and φ = 145 (2)°. The sum of bond angles around N1 [331.99 (3)°] indicates *sp*^3^ hybridization. The pyrrolidine ring makes dihedral angles of 54.47 (5)°, 51.50 (5)° and 73.37 (6)° with the napthalene,tetrahydro pyran and phenyl rings respectively. The napthalene and tetrahydro pyran rings are almost planar with each other with a dihedral angle of 8.88 (4)°,

In the crystal packing, atom O3 is involved in intramolecular C - H···O interactions and atom N1 contributes to C - H···N intramolecular interactions.

### Experimental

A mixture of (*Z*)-methyl 2-(1-formylnaphthalen-2-yloxy)-3-*p*-tolylacrylate and pipecolinic acid were refluxed in benzene for 20 h and the solvent was removed under reduced pressure. The crude product was subjected to column chromatography to get the pure product. The product was recrystallized from dry benzene by slow evaporation.

### Refinement

H atoms were placed in idealized positions and allowed to ride on their parent atoms, with C–H = 0.93 or 0.96 Å and Uĩso~(H)= 1.2–1.5U~eq~(C).

### Crystal data

**Table:**

C_27_H_27_NO_3_	*Z* = 2
*M**_r_* = 413.50	*F*(000) = 440
Triclinic, *P*1	*D*_x_ = 1.288 Mg m^−^^3^
Hall symbol: -P 1	Mo *K*α radiation, λ = 0.71073 Å
*a* = 9.4201 (3) Å	Cell parameters from 22685 reflections
*b* = 10.6752 (3) Å	θ = 2.0–27.0°
*c* = 11.0761 (3) Å	µ = 0.08 mm^−^^1^
α = 78.262 (2)°	*T* = 293 K
β = 77.911 (2)°	Needle, colourless
γ = 87.346 (2)°	0.30 × 0.20 × 0.15 mm
*V* = 1066.34 (5) Å^3^	

### Data collection

**Table:**

Bruker Kappa APEXII diffractometer	4641 independent reflections
Radiation source: fine-focus sealed tube	3461 reflections with *I* > 2σ(*I*)
graphite	*R*_int_ = 0.026
ω and φ scans	θ_max_ = 27.0°, θ_min_ = 2.0°
Absorption correction: multi-scan (*SADABS*; Sheldrick, 1996)	*h* = −11→12
*T*_min_ = 0.975, *T*_max_ = 0.988	*k* = −13→13
22685 measured reflections	*l* = −14→14

### Refinement

**Table:**

Refinement on *F*^2^	Secondary atom site location: difference Fourier map
Least-squares matrix: full	Hydrogen site location: inferred from neighbouring sites
*R*[*F*^2^ > 2σ(*F*^2^)] = 0.044	H-atom parameters constrained
*wR*(*F*^2^) = 0.122	*w* = 1/[σ^2^(*F*_o_^2^) + (0.0534*P*)^2^ + 0.3168*P*] where *P* = (*F*_o_^2^ + 2*F*_c_^2^)/3
*S* = 1.00	(Δ/σ)_max_ < 0.001
4641 reflections	Δρ_max_ = 0.34 e Å^−^^3^
281 parameters	Δρ_min_ = −0.23 e Å^−^^3^
0 restraints	Extinction correction: *SHELXL97* (Sheldrick, 2008), Fc^*^=kFc[1+0.001xFc^2^λ^3^/sin(2θ)]^-1/4^
Primary atom site location: structure-invariant direct methods	Extinction coefficient: 0.012 (2)

### Special details

**Table:**

Geometry. All e.s.d.'s (except the e.s.d. in the dihedral angle between two l.s. planes) are estimated using the full covariance matrix. The cell e.s.d.'s are taken into account individually in the estimation of e.s.d.'s in distances, angles and torsion angles; correlations between e.s.d.'s in cell parameters are only used when they are defined by crystal symmetry. An approximate (isotropic) treatment of cell e.s.d.'s is used for estimating e.s.d.'s involving l.s. planes.
Refinement. Refinement of *F*^2^ against ALL reflections. The weighted *R*-factor *wR* and goodness of fit *S* are based on *F*^2^, conventional *R*-factors *R* are based on *F*, with *F* set to zero for negative *F*^2^. The threshold expression of *F*^2^ > σ(*F*^2^) is used only for calculating *R*-factors(gt) *etc*. and is not relevant to the choice of reflections for refinement. *R*-factors based on *F*^2^ are statistically about twice as large as those based on *F*, and *R*- factors based on ALL data will be even larger.

### Fractional atomic coordinates and isotropic or equivalent isotropic displacement parameters (Å^2^)

**Table:**

	*x*	*y*	*z*	*U*_iso_*/*U*_eq_	
C1	0.54853 (15)	0.76212 (14)	0.13837 (13)	0.0396 (3)	
H1A	0.4898	0.7171	0.2165	0.047*	
H1B	0.5454	0.8527	0.1403	0.047*	
C2	0.70388 (16)	0.71426 (15)	0.12642 (15)	0.0453 (4)	
H2A	0.7052	0.6221	0.1343	0.054*	
H2B	0.7439	0.7339	0.1943	0.054*	
C3	0.79742 (17)	0.77508 (18)	0.00095 (16)	0.0540 (4)	
H3A	0.8930	0.7357	−0.0077	0.065*	
H3B	0.8089	0.8655	−0.0016	0.065*	
C4	0.72958 (17)	0.75893 (18)	−0.10770 (16)	0.0504 (4)	
H4A	0.7854	0.8056	−0.1864	0.060*	
H4B	0.7295	0.6692	−0.1125	0.060*	
C5	0.57534 (15)	0.80960 (13)	−0.08654 (13)	0.0366 (3)	
H5	0.5812	0.8987	−0.0780	0.044*	
C6	0.47933 (16)	0.80945 (13)	−0.18463 (13)	0.0378 (3)	
H6	0.4643	0.8996	−0.2206	0.045*	
C7	0.32917 (15)	0.76056 (13)	−0.10062 (13)	0.0365 (3)	
C8	0.33931 (14)	0.78431 (12)	0.02922 (13)	0.0340 (3)	
H8	0.3313	0.8762	0.0288	0.041*	
C9	0.22386 (15)	0.71384 (13)	0.13304 (13)	0.0376 (3)	
C10	0.17673 (15)	0.75355 (14)	0.25133 (14)	0.0412 (3)	
C11	0.23444 (18)	0.86035 (16)	0.28129 (15)	0.0476 (4)	
H11	0.3068	0.9082	0.2220	0.057*	
C12	0.1870 (2)	0.8954 (2)	0.39497 (17)	0.0612 (5)	
H12	0.2275	0.9661	0.4118	0.073*	
C13	0.0784 (2)	0.8262 (2)	0.48593 (17)	0.0714 (6)	
H13	0.0478	0.8495	0.5638	0.086*	
C14	0.0178 (2)	0.7247 (2)	0.46007 (17)	0.0658 (5)	
H14	−0.0558	0.6797	0.5205	0.079*	
C15	0.06357 (17)	0.68546 (16)	0.34362 (15)	0.0501 (4)	
C16	−0.00152 (18)	0.58123 (17)	0.31539 (17)	0.0572 (5)	
H16	−0.0748	0.5357	0.3758	0.069*	
C17	0.04018 (17)	0.54627 (16)	0.20290 (18)	0.0540 (4)	
H17	−0.0050	0.4782	0.1857	0.065*	
C18	0.15284 (16)	0.61359 (14)	0.11133 (15)	0.0431 (4)	
C19	0.30894 (17)	0.61779 (14)	−0.08800 (15)	0.0441 (4)	
H19A	0.3044	0.6000	−0.1696	0.053*	
H19B	0.3921	0.5724	−0.0612	0.053*	
C20	0.53277 (16)	0.74536 (14)	−0.29578 (14)	0.0413 (3)	
C21	0.59051 (17)	0.62247 (15)	−0.28796 (15)	0.0478 (4)	
H21	0.6021	0.5754	−0.2102	0.057*	
C22	0.63086 (19)	0.56903 (18)	−0.39354 (18)	0.0571 (4)	
H22	0.6691	0.4866	−0.3863	0.069*	
C23	0.6149 (2)	0.6367 (2)	−0.50911 (18)	0.0688 (5)	
H23	0.6415	0.6004	−0.5801	0.083*	
C24	0.5594 (2)	0.7582 (2)	−0.51919 (17)	0.0750 (6)	
H24	0.5486	0.8048	−0.5974	0.090*	
C25	0.5192 (2)	0.81199 (18)	−0.41372 (15)	0.0589 (5)	
H25	0.4823	0.8949	−0.4222	0.071*	
C26	0.20768 (16)	0.83093 (14)	−0.15768 (14)	0.0420 (4)	
C27	0.0803 (2)	1.02515 (18)	−0.1933 (2)	0.0728 (6)	
H27A	0.0737	1.1075	−0.1703	0.109*	
H27B	−0.0126	0.9839	−0.1656	0.109*	
H27C	0.1093	1.0355	−0.2832	0.109*	
N1	0.49017 (12)	0.74134 (10)	0.03267 (10)	0.0349 (3)	
O1	0.17989 (12)	0.57199 (10)	0.00045 (11)	0.0524 (3)	
O2	0.13962 (15)	0.78741 (13)	−0.21840 (14)	0.0738 (4)	
O3	0.18643 (13)	0.94760 (11)	−0.13447 (13)	0.0610 (4)	

### Atomic displacement parameters (Å^2^)

**Table:**

	*U*^11^	*U*^22^	*U*^33^	*U*^12^	*U*^13^	*U*^23^
C1	0.0406 (8)	0.0426 (8)	0.0360 (8)	0.0003 (6)	−0.0123 (6)	−0.0045 (6)
C2	0.0417 (8)	0.0485 (8)	0.0495 (9)	0.0022 (7)	−0.0186 (7)	−0.0092 (7)
C3	0.0359 (8)	0.0718 (11)	0.0571 (11)	−0.0019 (8)	−0.0116 (7)	−0.0172 (9)
C4	0.0384 (8)	0.0672 (10)	0.0466 (9)	−0.0003 (7)	−0.0068 (7)	−0.0153 (8)
C5	0.0401 (8)	0.0355 (7)	0.0331 (7)	−0.0035 (6)	−0.0068 (6)	−0.0042 (6)
C6	0.0427 (8)	0.0346 (7)	0.0344 (8)	0.0016 (6)	−0.0092 (6)	−0.0020 (6)
C7	0.0380 (7)	0.0355 (7)	0.0368 (8)	0.0032 (6)	−0.0112 (6)	−0.0062 (6)
C8	0.0354 (7)	0.0320 (6)	0.0348 (7)	0.0028 (5)	−0.0116 (6)	−0.0035 (5)
C9	0.0332 (7)	0.0377 (7)	0.0384 (8)	0.0052 (6)	−0.0093 (6)	0.0010 (6)
C10	0.0353 (7)	0.0467 (8)	0.0377 (8)	0.0105 (6)	−0.0105 (6)	0.0016 (6)
C11	0.0452 (9)	0.0583 (9)	0.0393 (8)	0.0089 (7)	−0.0116 (7)	−0.0083 (7)
C12	0.0594 (11)	0.0808 (13)	0.0485 (10)	0.0128 (9)	−0.0170 (9)	−0.0209 (9)
C13	0.0698 (13)	0.1044 (17)	0.0372 (10)	0.0179 (12)	−0.0085 (9)	−0.0145 (10)
C14	0.0548 (11)	0.0877 (14)	0.0406 (10)	0.0156 (10)	0.0001 (8)	0.0062 (9)
C15	0.0393 (8)	0.0583 (10)	0.0431 (9)	0.0113 (7)	−0.0065 (7)	0.0075 (7)
C16	0.0392 (9)	0.0582 (10)	0.0591 (11)	0.0025 (8)	0.0005 (8)	0.0119 (8)
C17	0.0380 (8)	0.0464 (9)	0.0707 (12)	−0.0031 (7)	−0.0078 (8)	0.0018 (8)
C18	0.0356 (7)	0.0408 (8)	0.0492 (9)	0.0031 (6)	−0.0088 (7)	−0.0013 (7)
C19	0.0437 (8)	0.0409 (8)	0.0480 (9)	−0.0014 (6)	−0.0076 (7)	−0.0113 (7)
C20	0.0406 (8)	0.0486 (8)	0.0342 (8)	−0.0004 (6)	−0.0088 (6)	−0.0058 (6)
C21	0.0476 (9)	0.0520 (9)	0.0443 (9)	0.0055 (7)	−0.0102 (7)	−0.0111 (7)
C22	0.0482 (9)	0.0654 (11)	0.0605 (11)	0.0050 (8)	−0.0059 (8)	−0.0255 (9)
C23	0.0632 (12)	0.0998 (16)	0.0486 (11)	0.0020 (11)	−0.0046 (9)	−0.0338 (11)
C24	0.0890 (15)	0.1004 (16)	0.0344 (10)	0.0088 (13)	−0.0153 (10)	−0.0098 (10)
C25	0.0689 (12)	0.0660 (11)	0.0385 (9)	0.0091 (9)	−0.0128 (8)	−0.0031 (8)
C26	0.0432 (8)	0.0482 (8)	0.0380 (8)	0.0029 (7)	−0.0154 (7)	−0.0098 (6)
C27	0.0742 (13)	0.0566 (11)	0.0990 (16)	0.0198 (9)	−0.0548 (12)	−0.0080 (10)
N1	0.0328 (6)	0.0373 (6)	0.0334 (6)	0.0007 (5)	−0.0090 (5)	−0.0022 (5)
O1	0.0482 (6)	0.0477 (6)	0.0609 (7)	−0.0112 (5)	−0.0050 (5)	−0.0140 (5)
O2	0.0823 (10)	0.0762 (9)	0.0850 (10)	0.0176 (7)	−0.0542 (8)	−0.0328 (8)
O3	0.0680 (8)	0.0456 (6)	0.0851 (9)	0.0183 (5)	−0.0509 (7)	−0.0167 (6)

### Geometric parameters (Å, °)

**Table:**

C1—N1	1.4521 (18)	C12—H12	0.9300
C1—C2	1.5157 (19)	C13—C14	1.355 (3)
C1—H1A	0.9700	C13—H13	0.9300
C1—H1B	0.9700	C14—C15	1.412 (3)
C2—C3	1.517 (2)	C14—H14	0.9300
C2—H2A	0.9700	C15—C16	1.413 (3)
C2—H2B	0.9700	C16—C17	1.348 (3)
C3—C4	1.518 (2)	C16—H16	0.9300
C3—H3A	0.9700	C17—C18	1.412 (2)
C3—H3B	0.9700	C17—H17	0.9300
C4—C5	1.515 (2)	C18—O1	1.3604 (19)
C4—H4A	0.9700	C19—O1	1.4276 (18)
C4—H4B	0.9700	C19—H19A	0.9700
C5—N1	1.4618 (17)	C19—H19B	0.9700
C5—C6	1.553 (2)	C20—C25	1.383 (2)
C5—H5	0.9800	C20—C21	1.390 (2)
C6—C20	1.5128 (19)	C21—C22	1.379 (2)
C6—C7	1.568 (2)	C21—H21	0.9300
C6—H6	0.9800	C22—C23	1.370 (3)
C7—C26	1.5178 (19)	C22—H22	0.9300
C7—C19	1.5191 (19)	C23—C24	1.368 (3)
C7—C8	1.5328 (19)	C23—H23	0.9300
C8—N1	1.4788 (16)	C24—C25	1.380 (3)
C8—C9	1.5070 (19)	C24—H24	0.9300
C8—H8	0.9800	C25—H25	0.9300
C9—C18	1.375 (2)	C26—O2	1.1892 (18)
C9—C10	1.435 (2)	C26—O3	1.3203 (18)
C10—C11	1.412 (2)	C27—O3	1.4445 (18)
C10—C15	1.422 (2)	C27—H27A	0.9600
C11—C12	1.366 (2)	C27—H27B	0.9600
C11—H11	0.9300	C27—H27C	0.9600
C12—C13	1.393 (3)		
			
N1—C1—C2	110.04 (12)	C11—C12—H12	119.7
N1—C1—H1A	109.7	C13—C12—H12	119.7
C2—C1—H1A	109.7	C14—C13—C12	119.46 (18)
N1—C1—H1B	109.7	C14—C13—H13	120.3
C2—C1—H1B	109.7	C12—C13—H13	120.3
H1A—C1—H1B	108.2	C13—C14—C15	121.63 (18)
C1—C2—C3	111.46 (13)	C13—C14—H14	119.2
C1—C2—H2A	109.3	C15—C14—H14	119.2
C3—C2—H2A	109.3	C14—C15—C16	121.75 (17)
C1—C2—H2B	109.3	C14—C15—C10	119.33 (18)
C3—C2—H2B	109.3	C16—C15—C10	118.91 (15)
H2A—C2—H2B	108.0	C17—C16—C15	121.48 (16)
C2—C3—C4	110.99 (14)	C17—C16—H16	119.3
C2—C3—H3A	109.4	C15—C16—H16	119.3
C4—C3—H3A	109.4	C16—C17—C18	119.65 (17)
C2—C3—H3B	109.4	C16—C17—H17	120.2
C4—C3—H3B	109.4	C18—C17—H17	120.2
H3A—C3—H3B	108.0	O1—C18—C9	124.15 (14)
C5—C4—C3	108.85 (13)	O1—C18—C17	113.58 (14)
C5—C4—H4A	109.9	C9—C18—C17	122.24 (15)
C3—C4—H4A	109.9	O1—C19—C7	111.98 (12)
C5—C4—H4B	109.9	O1—C19—H19A	109.2
C3—C4—H4B	109.9	C7—C19—H19A	109.2
H4A—C4—H4B	108.3	O1—C19—H19B	109.2
N1—C5—C4	110.61 (12)	C7—C19—H19B	109.2
N1—C5—C6	104.91 (11)	H19A—C19—H19B	107.9
C4—C5—C6	120.75 (12)	C25—C20—C21	117.25 (15)
N1—C5—H5	106.6	C25—C20—C6	117.84 (14)
C4—C5—H5	106.6	C21—C20—C6	124.88 (13)
C6—C5—H5	106.6	C22—C21—C20	121.16 (16)
C20—C6—C5	120.22 (12)	C22—C21—H21	119.4
C20—C6—C7	115.04 (12)	C20—C21—H21	119.4
C5—C6—C7	103.10 (11)	C23—C22—C21	120.37 (17)
C20—C6—H6	105.8	C23—C22—H22	119.8
C5—C6—H6	105.8	C21—C22—H22	119.8
C7—C6—H6	105.8	C24—C23—C22	119.46 (17)
C26—C7—C19	108.51 (12)	C24—C23—H23	120.3
C26—C7—C8	115.68 (11)	C22—C23—H23	120.3
C19—C7—C8	107.65 (11)	C23—C24—C25	120.25 (18)
C26—C7—C6	109.40 (11)	C23—C24—H24	119.9
C19—C7—C6	112.66 (11)	C25—C24—H24	119.9
C8—C7—C6	102.97 (11)	C24—C25—C20	121.49 (18)
N1—C8—C9	115.59 (10)	C24—C25—H25	119.3
N1—C8—C7	99.80 (10)	C20—C25—H25	119.3
C9—C8—C7	112.33 (12)	O2—C26—O3	123.07 (14)
N1—C8—H8	109.6	O2—C26—C7	124.22 (14)
C9—C8—H8	109.6	O3—C26—C7	112.70 (12)
C7—C8—H8	109.6	O3—C27—H27A	109.5
C18—C9—C10	118.14 (13)	O3—C27—H27B	109.5
C18—C9—C8	119.29 (13)	H27A—C27—H27B	109.5
C10—C9—C8	122.39 (13)	O3—C27—H27C	109.5
C11—C10—C15	117.06 (15)	H27A—C27—H27C	109.5
C11—C10—C9	123.40 (14)	H27B—C27—H27C	109.5
C15—C10—C9	119.52 (15)	C1—N1—C5	110.91 (11)
C12—C11—C10	121.81 (17)	C1—N1—C8	116.93 (11)
C12—C11—H11	119.1	C5—N1—C8	104.15 (10)
C10—C11—H11	119.1	C18—O1—C19	116.88 (12)
C11—C12—C13	120.7 (2)	C26—O3—C27	116.46 (13)

### Hydrogen-bond geometry (Å, °)

**Table:**

*D*—H···*A*	*D*—H	H···*A*	*D*···*A*	*D*—H···*A*
C8—H8···O3	0.98	2.47	2.8240 (19)	101
C19—H19B···N1	0.97	2.55	2.885 (2)	100
